# Publisher Correction: Identification and structure of an extracellular contractile injection system from the marine bacterium *Algoriphagus machipongonensis*

**DOI:** 10.1038/s41564-022-01111-1

**Published:** 2022-04-04

**Authors:** Jingwei Xu, Charles F. Ericson, Yun-Wei Lien, Florentine U. N. Rutaganira, Fabian Eisenstein, Miki Feldmüller, Nicole King, Martin Pilhofer

**Affiliations:** 1 Department of Biology, Institute of Molecular Biology & Biophysics, Eidgenössische Technische Hochschule Zürich, Otto-Stern-Weg 5, Zürich, Switzerland; 2 Howard Hughes Medical Institute and Department of Molecular and Cell Biology, University of California, Berkeley, CA USA; 3 Present Address: Graduate School of Medicine, University of Tokyo, N415, 7-3-1 Hongo, Bunkyo-ku, Tokyo, Japan

**Keywords:** Bacterial structural biology, Electron microscopy

Correction to: *Nature Microbiology* 10.1038/s41564-022-01059-2, published online 14 February 2022.

In the version of this article initially published, in the first sentence of the last paragraph of the “Generation of AlgoCIS mutants” section in the Methods now reading “The in-frame deletions of the AlgoCIS genes were generated utilizing our pCHIP3 suicide plasmid (Extended Data Fig. 4e),” Extended Data Fig. 5e was originally cited in error. Further, ref. 49 has been updated with complete publication information, as below. The errors have been corrected in the HTML and PDF versions of the article.

49. Weiss, G. L. et al. Structure of a thylakoid-anchored contractile injection system in multicellular cyanobacteria. *Nat. Microbiol*. 10.1038/s41564-021-01055-y (2022).

