# Supplementary material for: Identification and structure of an extracellular contractile injection system from the marine bacterium Algoriphagus machipongonensis
Source: Nat Microbiol. 2022 Feb 14;7(3):397–410. doi: 10.1038/s41564-022-01059-2 (PMC8894135; doi:10.1038/s41564-022-01059-2)
Supplement: Supplementary file 1 — Supplementary Figs. 1–3 and Tables 1–4. [file 41564_2022_1059_MOESM1_ESM.pdf]

nature portfolio

https://doi.org/10.1038/s41564-022-01059-2

Supplementary information

Identification and structure of an extracellular contractile injection system from the marine bacterium *Algoriphagus machipongonensis*

In the format provided by the authors and unedited

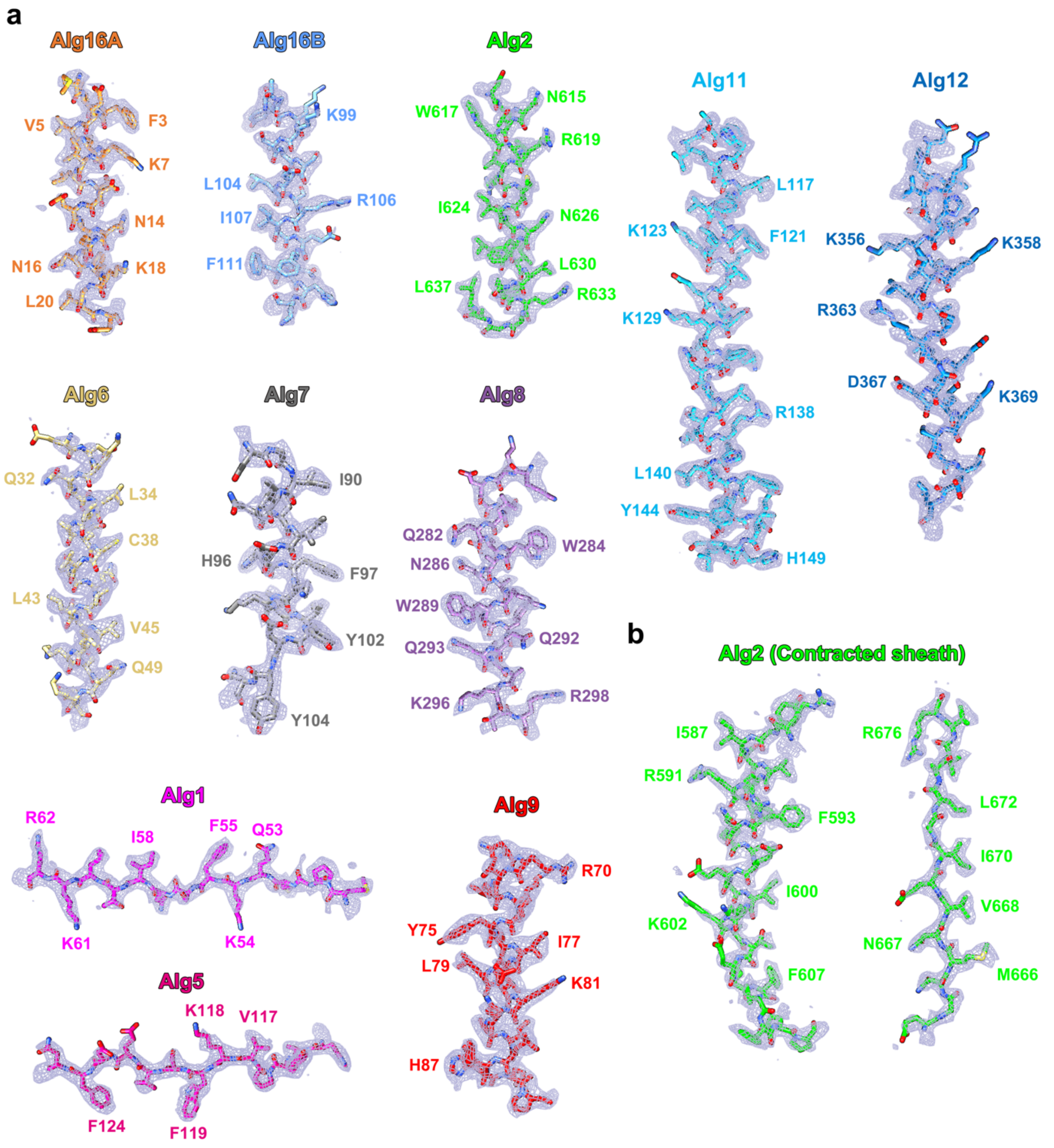

Supplementary Figure 1. Representative densities of reconstructed EM maps

a: Stick and density mesh diagrams showing the representative density maps of different structural components in the extended AlgoCIS particles. The color code for different proteins matches Fig. 1d, while the density meshes are colored grey.

b: Stick and density mesh diagrams showing the representative density maps of putative sheath protein (Alg2) in the contracted sheath. The color code for Alg2 matches Fig. 1d, while the density meshes are colored grey.

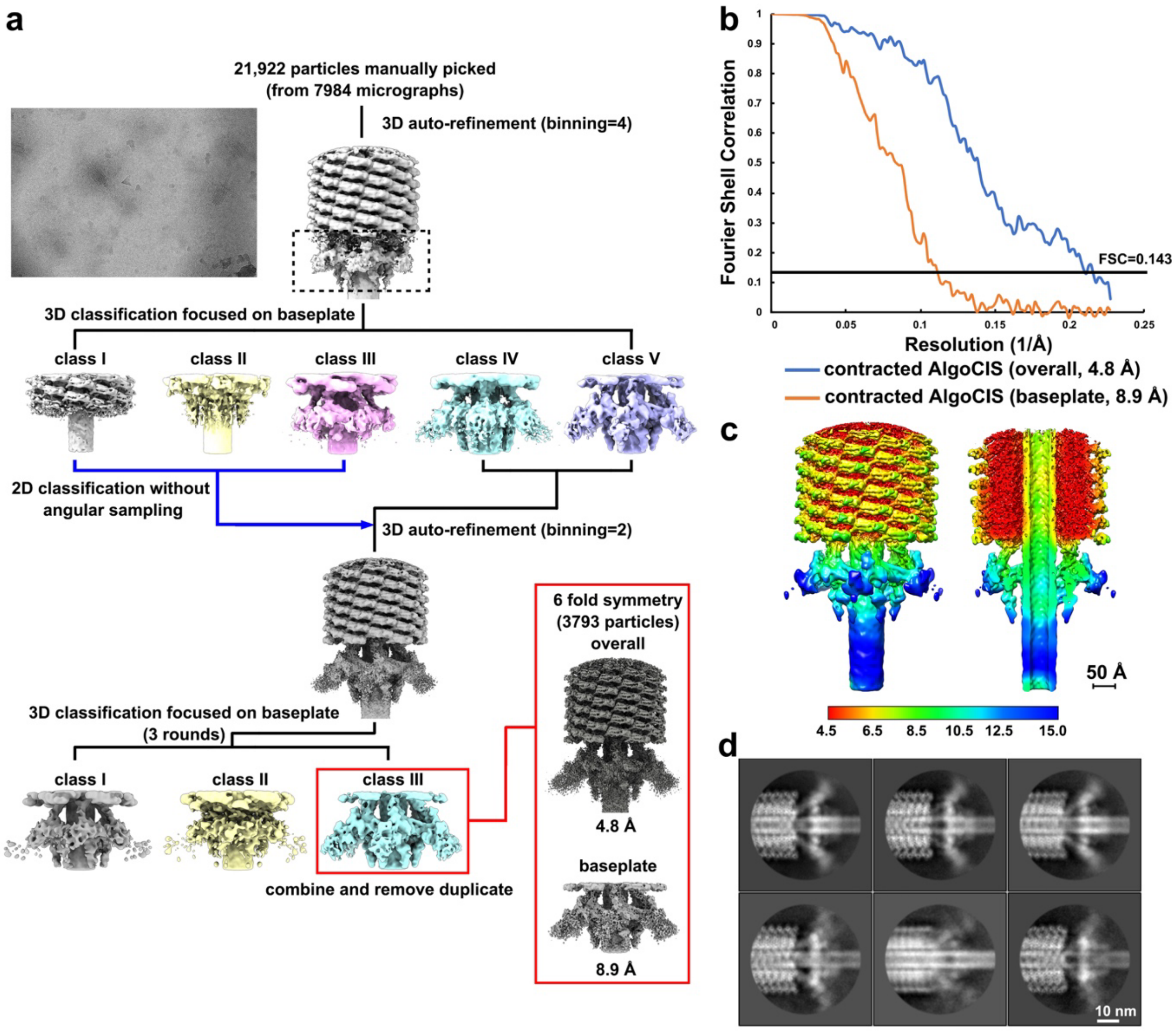

Supplementary Figure 2. Workflow for the cryoEM structural determinations of the contracted AlgoCIS baseplate

a: Flowchart for cryoEM reconstruction of the contracted AlgoCIS baseplate. See METHODS and Supplementary Table 3 for details.

b: Gold standard FSC curves of the contracted AlgoCIS baseplate reconstructions, including the overall structure (blue) and the focused baseplate structure (orange).

c: Local resolution maps of the overall structure of the contracted AlgoCIS baseplate.

d: 2D classification analyses showing the empty lumen of the expelled inner tube in the contracted AlgoCIS.

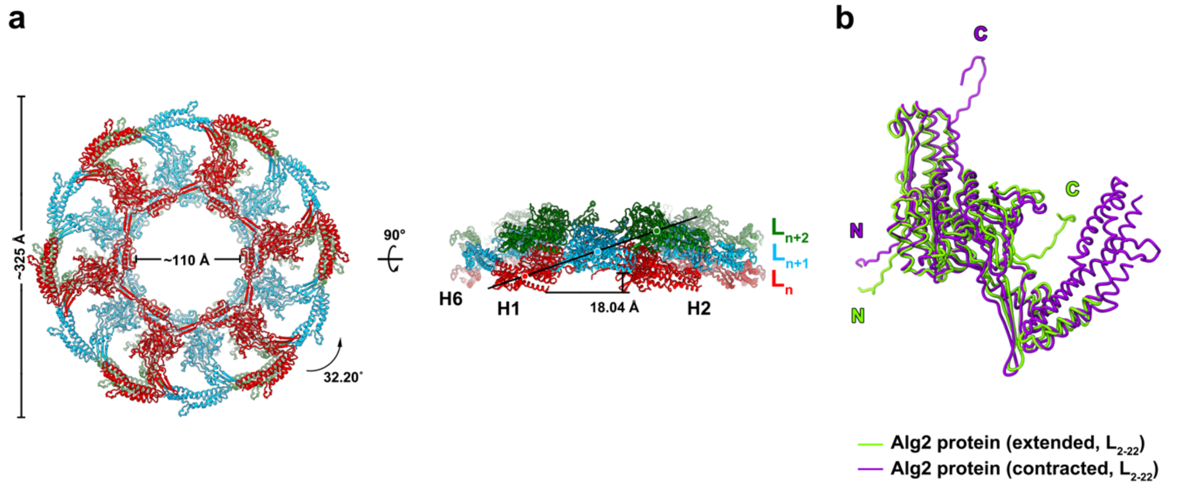

Supplementary Figure 3. CryoEM structures of the contracted AlgoCIS sheath fragment

a: Top view (left) and side view (right) of ribbon diagrams showing the contracted sheath fragment containing three sheath layers (L_n, L_{n+1} and L_{n+2}). The protein layers are represented in different colors. The direction of one helical strand (H1) is highlighted by a line.

b: Structural superpositions of sheath subunits in the extended (green) and contracted (purple) states showing that the displacements of N- and C-termini of Alg2 mediate a rigid body rotation of the subunit.

Supplementary Table 1. AlgoCIS gene cluster

	Accession number	Protein length	Number of copies	Description	Homolog in AFP	Homolog in PVC	Homolog in T4
Alg16A	A3HTC4	197	6	Cap terminator	Afp16	Pvc16	gp15
Alg16B	A3HTC3	284	6	Adaptor between cap terminator and sheath-tube module	-	-	-
Alg2	A3HTC2	692	138	sheath protein	Afp2/3/4	Pvc2/3/4	gp18
Alg1	A3HTC1	142	132	inner tube protein	Afp1	Pvc1	gp19
Alg5	A3HTC0	147	6	tube initiator	Afp5	Pvc5	gp54
Alg6	A3HTB9	52	3	plugged protein at the proximal end of the inner tube lumen	Afp6	Pvc6	-
Alg7	A3HTB8	228	6	tube initiator, LysM-like protein	Afp7	Pvc7	gp48/gp53
Alg8	A3HTB7	581	3	Baseplate, VgrG-like protein	Afp8	Pvc8	gp5/gp27
Alg10	A3HTB6	96	1	Baseplate, PAAR-like protein	Afp10	Pvc10	gp5.4
Alg9	A3HTB5	137	6	Baseplate, wedge protein	Afp9	Pvc9	gp25
Alg11	A3HTB4	1050	6	Baseplate, wedge protein	Afp11	Pvc11	gp6
Alg12	A3HTB3	933	6	Baseplate, wedge protein	Afp12	Pvc12	gp6/gp7
Alg19	A3HTB2	1443	unknown	Ig-like fold protein, potential tail fiber	-	-	-
Alg14	A3HTB1	537	-	potential tape measure protein	Afp14	Pvc14	gp29
ALPR1_12695 (Cgo1)	A3HTB0	498	unknown	DUF4157 containing protein (cargo protein)	-	-	-
ALPR1_12690 (Cgo2)	A3HTA9	1228	unknown	DUF4157 containing protein (cargo protein)	-	-	-
ALPR1_12685	A3HTA8	289	-	tetratricopeptide repeat protein	-	-	-
Alg15	A3HTA7	435	-	AAA superfamily ATPase	Afp15	Pvc15	-

Supplementary Table 2. Mass spectrum analysis of different purified samples of AlgoCIS wild-type and the corresponding mutants.

	Purified extended AlgoCIS sample	Purified contracted AlgoCIS sample	Sample from WT culture supernatant	Sample from null mutant culture supernatant	Sample from AlgoCIS⁻ culture supernatant	AlgoCIS Cgo1/Cgo2⁻	AlgoCIS Cgo1⁻	AlgoCIS Cgo2⁻	AlgoCIS ΔAlg16B
Agl16A	10	8	9	8	-	24	-	1	5
Alg16B	19	11	13	14	-	36	21	9	-
Alg2	33	38	26	25	3	141	54	27	35
Alg1	13	12	4	2	-	17	2	1	14
Alg5	6	6	-	-	-	-	-	-	5
Alg6	2	-	-	-	-	-	4	-	3
Alg7	16	13	9	5	-	12	5	5	7
Alg8	19	4	12	15	-	35	22	15	20
Alg10	-	-	-	-	-	-	-	-	3
Agl9	6	2	4	3	-	9	4	4	7
Alg11	37	32	24	30	-	115	42	24	52
Alg12	40	37	30	34	-	117	21	8	70
Agl19	69	59	58	61	-	85	95	39	86
Alg14	-	-	-	-	-	-	-	-	3
ALPR1_126 95 (Cgo1)	11	-	5	10	-	4	-	14	-
ALPR1_126 90 (Cgo2)	28	4	15	15	-	5	57	-	-
ALPR1_126 85	-	-	-	-	-	-	-	-	-
Alg15	-	-	-	-	-	-	-	-	3

Supplementary Table 3. Cryo-EM data statistical analysis

	Cap	Baseplate_C6 (overall)	Baseplate_C6 (focused refined)	baseplate_C3	Extended sheath-tube module	Contracted sheath shell	Baseplate in contracted AlgoCIS
**Data collection and processing**							
Magnification	81,000						
Voltage (kV)	300						
Electron exposure (e^{-}/Å)	60 e^{-}/dose weighting (K3 camera)						
Defocus range (μm)	1.0-3.0						
Pixel size (Å/pixel)	1.1						2.2 (binning=2)
Symmetry imposed	C6	C6	C6	C3	C6 + helical (twist = 20.54˚, rise = 40.80 Å)	C6 + helical (twist = 32.20˚, rise = 18.04 Å)	C6
Initial particles (No.)	128473	128473	128473	128473	856113	157909	21922
Final particles (No.)	65059	82969	30211	82969	225305	92922	3793
Map resolution	2.5	2.7	2.9	2.8	2.4	2.5	4.8 (overall) 8.9 (baseplate)
FSC threshold	0.143	0.143	0.143	0.143	0.143	0.143	0.143
**Refinement**							
Map sharpening B factor (Å^2)	-40	-52	-33	-56	-60	-64	15 (overall) -22 (baseplate)
Model composition					(three layers)	(three layers)	
Non-hydrogen atoms	67800	135188		149562	92718	91008	-
Protein residues	8508	17155		19014	11736	11808	-
Chains	30	42		48	36	18	-
R.M.S deviations							
Bond length (Å)	0.015	0.014		0.015	0.015	0.005	-
Bond angles (˚)	1.549	1.514		1.582	1.674	0.875	-
Validation							
MolProbity score	1.21	1.23		1.31	1.20	1.02	-
Clashscore	4.30	3.55		4.57	4.17	1.82	-
Poor rotamer (%)	0.00	0.00		0.00	0.00	0.00	-
Ramachandran plot							
Favored (%)	98.03	97.58		97.62	98.61	97.61	-
Allowed (%)	1.97	2.42		2.38	1.39	2.39	-
Outlier (%)	0.00	0.00		0.00	0.00	0.00	-
Masked CC	0.85	0.80	0.82	0.78	0.81	0.86	-

Supplementary Table 4. List of primers to generate different bacterial mutants

ΔAlg16B	ΔAlg16B_dA	5'-GTTAAAAAGGATCGATCCTCTAGATGCATTCGTACAAAGCAAAAG-3'
	ΔAlg16B_dB	5'-GTTGCCATAGTAAAAAAGGTAAATTATAGCGTTTCGTAGT-3'
	ΔAlg16B_dC	5'-ACTACGAAACGCTATAATTTACCTTTTTTACTATGGCAAC-3'
	ΔAlg16B_dD	5'-CGTGAATTCAAAGGGAGAGCTCAGGAAGCAATGGCTGAATTG-3'
	Alg16B_SeqF	5'-GGGAAGAGGTCACGATGAAA-3'
	Alg16B_SeqR	5'-ATCGAGCAACGATGAAATCC-3'
ΔAlg2	ΔAlg2_dA	5'-GTTAAAAAGGATCGATCCTCTAGATGGGGGACTTTAGGAGGAAG-3'
	ΔAlg2_dB	5'-GTCTAGGTCAAGCTGCTTTAAGTAAAAAAGGTTTAAATGA-3'
	ΔAlg2_dC	5'-TCATTTAAACCTTTTTTACTTAAAGCAGCTTGACCTAGAC-3'
	ΔAlg2_dD	5'-CGTGAATTCAAAGGGAGAGCTCACACATTGAGCAACGAGCTG-3'
	Alg2_SeqF	5'-AGGTGGTGTATTGGGGATCA-3'
	Alg2_SeqR	5'-TTCCCGGCATTTTTATTTTG-3'
ΔAlg6	ΔAlg6_dA	5'-GTTAAAAAGGATCGATCCTCTAGATTGGAGGTCTGCTGCTTTTT-3'
	ΔAlg6_dB	5'-CCAGTTTACCTTCACTCATGACCAGACAATCGTTTTGAAATAG-3'
	ΔAlg6_dC	5'-CTATTTCAAAACGATTGTCTGGTCATGAGTGAAGGTAAACTGG-3'
	ΔAlg6_dD	5'-CGTGAATTCAAAGGGAGAGCTCTCGTGACTCCACGATTAGGA-3'
	Alg6_SeqF	5'-TTTGCAGAGGGAGGAGAAAA-3'
	Alg6_SeqR	5'-GTCGTTGCCATGGCTCTAAT-3'
pCHIP3 backbone	pCHIP3_SeqF1	5'-TTCTGTTGCATGGGCATAAA-3'
	pCHIP3_SeqR1	5'-CCAACCCATCCATATGCTTC-3'
	pCHIP3_SeqR2	5'-CGTGAATTCAAAGGGAGAGC-3'
AlgoCIS^{-}	BamHI_Algo_CIS_F	5'-ATATATGGATCCTTTCTTTCATGCATAGG-3'
	Algo_CIS_F	5'-TCATTTAAACCTTTTTTACTTAAAGCAGCTTGACCTA-3'
	Algo_CIS_R	5'-TAGGTCAAGCTGCTTTAAGTAAAAAAGGTTTAAATGA-3'
	PstI_Algo_CIS_R	5'-ATATATCTGCAGTCGTATTGAACTAGTTCA-3'
Null mutant	BamHI_Algo_null_F	5'-ATATATGGATCCTGCTTCGATCTGCGCTTC-3'
	PstI_Algo_null_R	5'-ATATATCTGCAGCTCAAAAAAGCAGAAGCTCAG-3'
