# Supplementary material for: Identification and structure of an extracellular contractile injection system from the marine bacterium Algoriphagus machipongonensis
Source: Nat Microbiol. 2022 Feb 14;7(3):397–410. doi: 10.1038/s41564-022-01059-2 (PMC8894135; doi:10.1038/s41564-022-01059-2)
Supplement: Supplementary file 2 — Reporting Summary. [file 41564_2022_1059_MOESM2_ESM.pdf]

nature portfolio

Corresponding author(s): Martin Pilhofer
Last updated by author(s): Dec 13, 2021

Reporting Summary

Nature Portfolio wishes to improve the reproducibility of the work that we publish. This form provides structure for consistency and transparency in reporting. For further information on Nature Portfolio policies, see our Editorial Policies and the Editorial Policy Checklist.

Statistics

For all statistical analyses, confirm that the following items are present in the figure legend, table legend, main text, or Methods section.

n/a	Confirmed	
☐	☒	The exact sample size (*n*) for each experimental group/condition, given as a discrete number and unit of measurement
☐	☒	A statement on whether measurements were taken from distinct samples or whether the same sample was measured repeatedly
☒	☐	The statistical test(s) used AND whether they are one- or two-sided<br>*Only common tests should be described solely by name; describe more complex techniques in the Methods section.*
☒	☐	A description of all covariates tested
☒	☐	A description of any assumptions or corrections, such as tests of normality and adjustment for multiple comparisons
☐	☒	A full description of the statistical parameters including central tendency (e.g. means) or other basic estimates (e.g. regression coefficient) AND variation (e.g. standard deviation) or associated estimates of uncertainty (e.g. confidence intervals)
☒	☐	For null hypothesis testing, the test statistic (e.g. *F*, *t*, *r*) with confidence intervals, effect sizes, degrees of freedom and *P* value noted<br>*Give P values as exact values whenever suitable.*
☒	☐	For Bayesian analysis, information on the choice of priors and Markov chain Monte Carlo settings
☒	☐	For hierarchical and complex designs, identification of the appropriate level for tests and full reporting of outcomes
☒	☐	Estimates of effect sizes (e.g. Cohen's *d*, Pearson's *r*), indicating how they were calculated

Our web collection on statistics for biologists contains articles on many of the points above.

Software and code

Policy information about availability of computer code

Data collection	SerialEM-3.8.6 was used for cryo-EM/cryo-ET data collection, Metamorph (Version 7.7.11.0) for fluorescent light microscopy imaging
Data analysis	MotionCor2_1.1.0, Gctf-v1.06_sm_30, Relion-3.0 (including relion_helix_toolbox), UCSF Chimera-1.13, UCSF ChimeraX-1.1, COOT-0.8.9.1, PHENIX-1.13-2998 (including phenix.real_space_refine, phenix.molprobity), ImageJ-1.53f51(Fiji), IMOD-4.11.0, Rosetta-2018.09.60072, diffmap_120330, MEGAX, Prism 8.0, tom_deconv, MUSCLE, ESPript3, Dynamo-1.1.401

For manuscripts utilizing custom algorithms or software that are central to the research but not yet described in published literature, software must be made available to editors and reviewers. We strongly encourage code deposition in a community repository (e.g. GitHub). See the Nature Portfolio guidelines for submitting code & software for further information.

Data

Policy information about availability of data

All manuscripts must include a data availability statement. This statement should provide the following information, where applicable:

- Accession codes, unique identifiers, or web links for publicly available datasets
- A description of any restrictions on data availability
- For clinical datasets or third party data, please ensure that the statement adheres to our policy

The cryoEM density maps and corresponding atomic models have been deposited in the EMDB and PDB, respectively. The accession numbers are listed as following: cap module of AlgoCIS (PDB: 7ADZ and EMD-11734); sheath-tube module in the extended state (PDB: 7AE0 and EMD-11735); baseplate reconstructed in C6 symmetry (EMD-11743); baseplate focused refinement in C6 symmetry (PDB: 7AEB and EMD-11744); baseplate reconstructed in C3 symmetry (PDB: 7AEF and EMD-11745); overall AlgoCIS reconstruction (EMD-11746); sheath-tube module in the contracted state (PDB: 7AEK and EMD-11747); baseplate in the contracted state (EMD-11748). The representative reconstructed tomograms and the sub-tomogram averages have been deposited in the EMDB. The accession numbers are

listed below: sub-tomogram average of purified AlgoCIS (EMD-11749); sub-tomogram average of purified AlgoCIS Cgo1/Cgo2— mutant (EMD-11750); sub-tomogram average of purified AlgoCIS ΔAlg16B (EMD-13723); sub-tomogram average of baseplate in MAC arrays (EMD-13724); sub-tomogram average of baseplate in A. asiaticus (EMD-13725); in situ reconstructed tomogram of A. machipongonensis in bacterial late stage (EMD-13705); reconstructed tomogram of purified AlgoCIS (EMD-13722); reconstructed tomogram of purified AlgoCIS Cgo1/Cgo2— mutant (EMD-13719); reconstructed tomogram of purified AlgoCIS Cgo1— mutant (EMD-13720); reconstructed tomogram of purified AlgoCIS Cgo2— mutant (EMD-13721); reconstructed tomogram of purified AlgoCIS ΔAlg16B (EMD-13704).
The corresponding PDB entries (1OF4, 4JX0, 4ZXE, 5MWN, 6J0M, 6J0F, 6J0B, 6J0N, 6RBN, 6RBK, 6RAP, 6RAO) were used for the structural superpositions in the manuscript. The EMDB entries (EMD-9763, EMD-4876) were lowpass filtered and were used as initial reference in the structural determination procedure.
The raw numerical data about Fig. 2d, 5a, ED9c, and ED10d are included in the source data. The newick files for the phylogenetic trees in Fig. 1b/6a are provided in the source data.
Due to the size of the reconstructed tomograms of different AlgoCIS mutants, all tomograms are available from the authors with reasonable requests.

Field-specific reporting

Please select the one below that is the best fit for your research. If you are not sure, read the appropriate sections before making your selection.

☒ Life sciences ☐ Behavioural & social sciences ☐ Ecological, evolutionary & environmental sciences

For a reference copy of the document with all sections, see nature.com/documents/nr-reporting-summary-flat.pdf

Life sciences study design

All studies must disclose on these points even when the disclosure is negative.

Sample size	Sample size was stated in the text for each of our functional assays.
Data exclusions	No data was excluded from the analyses preformed.
Replication	All data was confirmed with proper biological replicates to ensure reproducibility of the assays shown in the study. Biological replicates of both the eukaryotic as well as the bacteria or sample preparations were used were the cultures were grown from frozen stock to readout independently of the related replicates.
Randomization	Assays in the study did not need to be randomized. Eukaryotic cells were grown up in appropriate growth media before being quantified to allow for the same number of cells to be placed into each well for the assays to keep the experiments consistent.
Blinding	For all experiments, blinding was not performed.

Reporting for specific materials, systems and methods

We require information from authors about some types of materials, experimental systems and methods used in many studies. Here, indicate whether each material, system or method listed is relevant to your study. If you are not sure if a list item applies to your research, read the appropriate section before selecting a response.

Materials & experimental systems

n/a	Involved in the study	
☐	☒	Antibodies
☐	☒	Eukaryotic cell lines
☒	☐	Palaeontology and archaeology
☒	☐	Animals and other organisms
☒	☐	Human research participants
☒	☐	Clinical data
☒	☐	Dual use research of concern

Methods

n/a	Involved in the study	
☒	☐	ChIP-seq
☒	☐	Flow cytometry
☒	☐	MRI-based neuroimaging

Antibodies

Antibodies used	Rabbit polyclonal anti-Alg2 (sheath protein, generated from GenScript); Rabbit polyclonal anti-Alg1 (inner tube protein, generated from GenScript); Rabbit polyclonal anti-RecA (abcam, ab63797)
Validation	The Rabbit polyclonal anti-RecA (ab63797) was reported to react with E. coli and be suitable for WB, IP based on the corresponding information in the website (https://www.abcam.com/reca-antibody-ab63797.html).<br>The qualities of rabbit polyclonal anti-Alg1 (inner tube protein) and anti-Alg2 (sheath protein) were tested by ELISA and WB with the purified antigen proteins in quality control part from GenScript company.

Eukaryotic cell lines

Policy information about cell lines

Cell line source(s)	Sf9 insect cells were from thermo fisher; J774A.1 murine macrophage line was purchased from ATCC; Choanoflagellate Salpingoeca rosetta culture with prey bacteria Echinicola pacifica (SrEpac) was from Nicola King's lab, which was also deposited at ATCC (PRA-390); Dictyostelium discoidum AX2 was from H. Hilbi lab; Acanthamoeba castellanii strain 5a2 was from lab stock, which was used in previous publication (Böck, D. et al. In situ architecture, function, and evolution of a contractile injection system. Sci New York N Y 357, 713–717 (2017))
Authentication	None of the cell lines were authenticated.
Mycoplasma contamination	Cell lines were not tested for Mycoplasma contamination
Commonly misidentified lines (See ICLAC register)	None.
