# Supplementary material for: Identification and structure of an extracellular contractile injection system from the marine bacterium Algoriphagus machipongonensis
Source: Nat Microbiol. 2022 Feb 14;7(3):397–410. doi: 10.1038/s41564-022-01059-2 (PMC8894135; doi:10.1038/s41564-022-01059-2)

Unprocessed western blot against Alg1 (inner tube protein) in Extended Data Fig. 1d

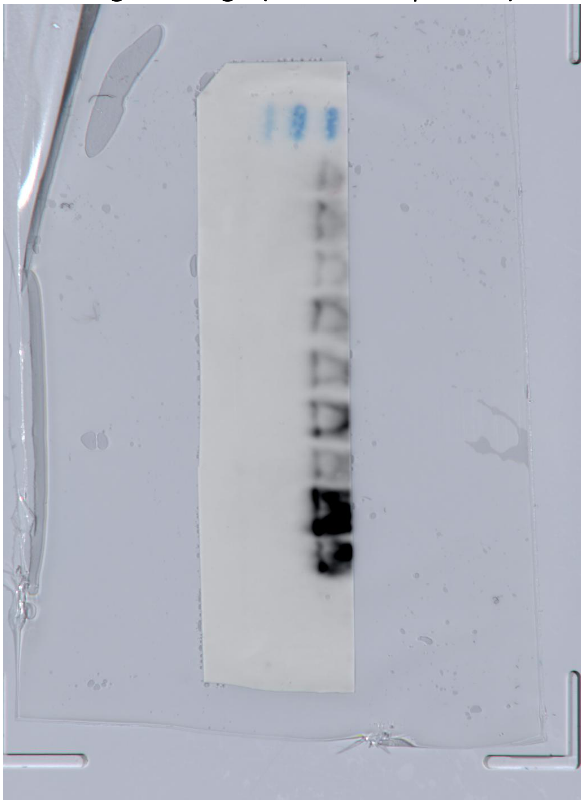

Unprocessed western blot against recA (loading control) in Extended Data Fig. 1d

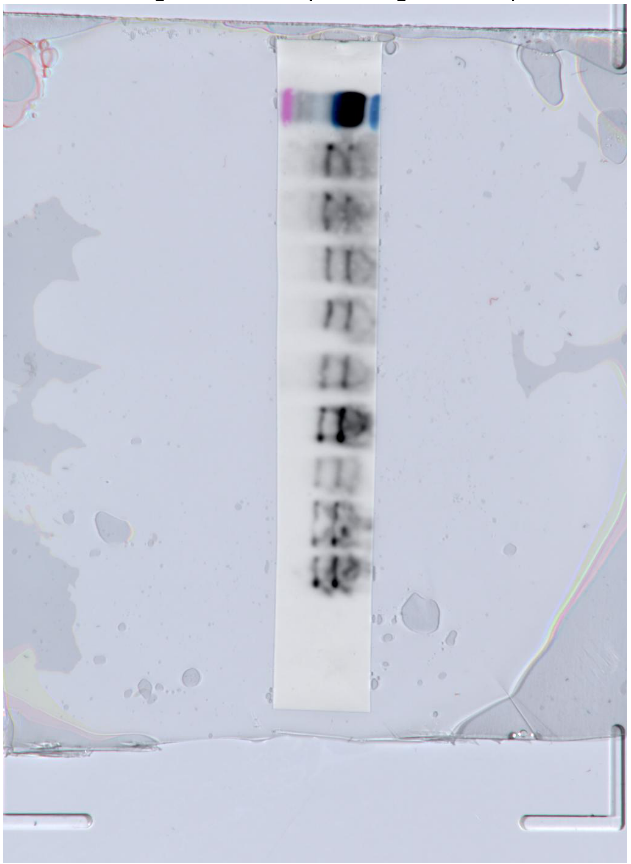

Unprocessed western blot against Alg1 (inner tube protein) in Extended Data Fig. 1e

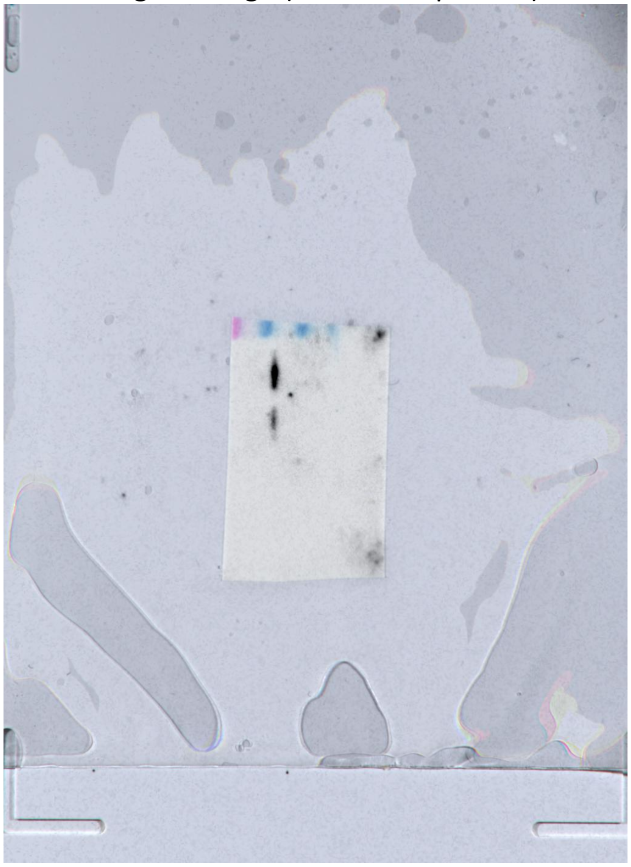

Unprocessed western blot against Alg2 (sheath protein) in Extended Data Fig. 1e

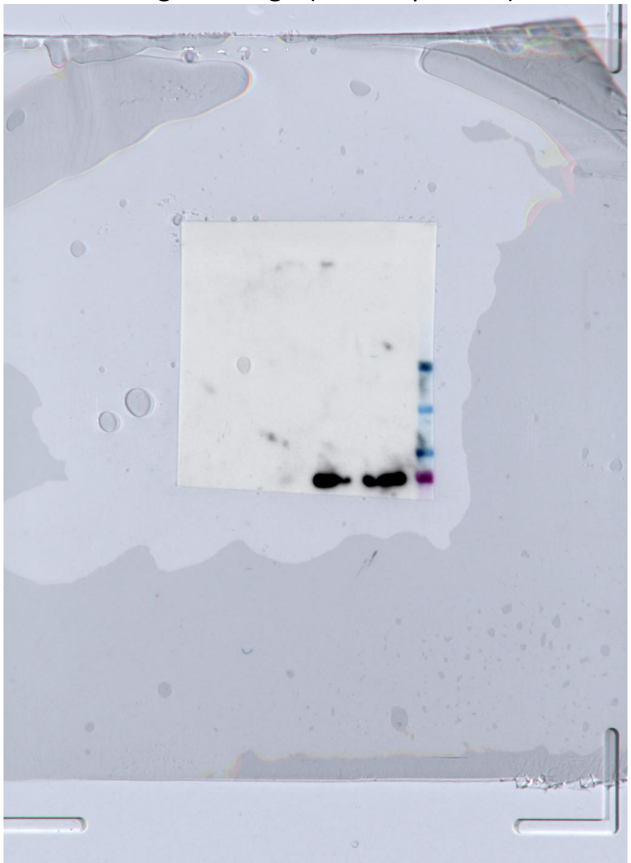

Unprocessed western blot against recA in Extended Data Fig. 1e

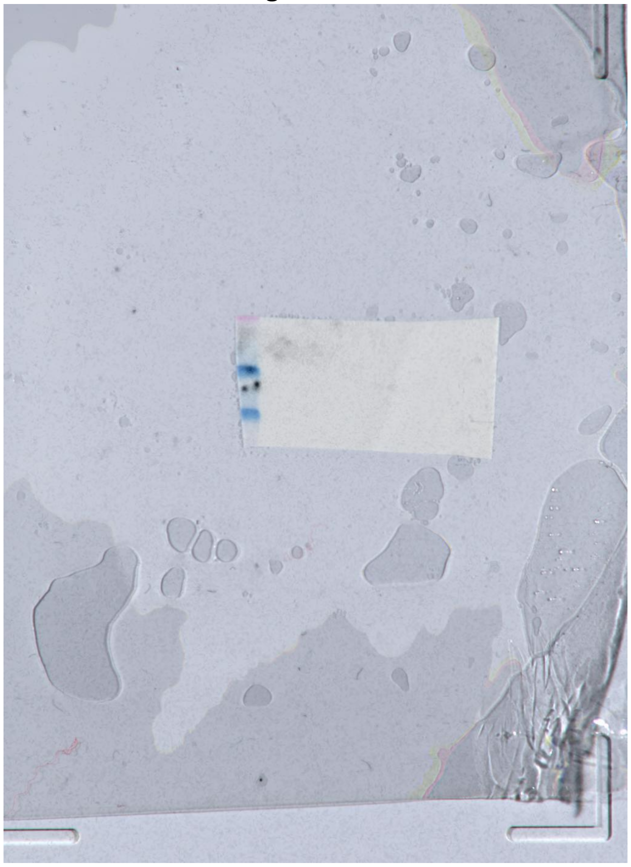

Supplement: Source Data Extended Data Fig. 1 — Unprocessed western blots for Extended Data Fig. 1d,e. [file 41564_2022_1059_MOESM9_ESM.pdf]
